# A Review on Zoonotic Pathogens Associated with Non-Human Primates: Understanding the Potential Threats to Humans

**DOI:** 10.3390/microorganisms11020246

**Published:** 2023-01-18

**Authors:** Xinjie Jiang, Zhenyu Fan, Shijia Li, Haichang Yin

**Affiliations:** 1 College of Life Science and Agriculture Forestry, Qiqihar University, Qiqihar 161006, China; 2 Heilongjiang Provincial Technology Innovation Center of Agromicrobial Preparation Industrialization, Qiqihar 161006, China; 3 Heilongjiang Provincial Key Laboratory of Resistance Gene Engineering and Protection of Biodiversity in Cold Areas, Qiqihar 161006, China

**Keywords:** non-human primate, viral pathogen, prevention measures, treatment measures, diagnostic methods, spread limitation

## Abstract

Non-human primates (NHP) share a close relationship with humans due to a genetic homology of 75–98.5%. NHP and humans have highly similar tissue structures, immunity, physiology, and metabolism and thus often can act as hosts to the same pathogens. Agriculture, meat consumption habits, tourism development, religious beliefs, and biological research have led to more extensive and frequent contact between NHPs and humans. Deadly viruses, such as rabies virus, herpes B virus, Marburg virus, Ebola virus, human immunodeficiency virus, and monkeypox virus can be transferred from NHP to humans. Similarly, herpes simplex virus, influenza virus, and yellow fever virus can be transmitted to NHP from humans. Infectious pathogens, including viruses, bacteria, and parasites, can affect the health of both primates and humans. A vast number of NHP-carrying pathogens exhibit a risk of transmission to humans. Therefore, zoonotic infectious diseases should be evaluated in future research. This article reviews the research evidence, diagnostic methods, prevention, and treatment measures that may be useful in limiting the spread of several common viral pathogens via NHP and providing ideas for preventing zoonotic diseases with epidemic potential.

## 1. Introduction

**Figure 1:**
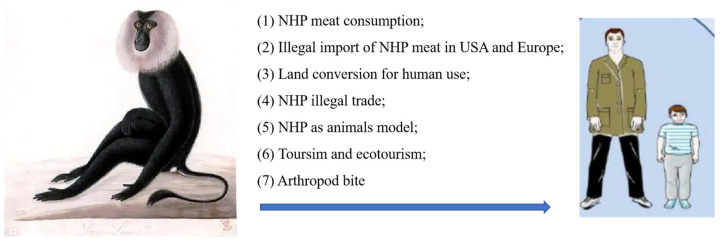
Drivers of interspecies pathogen transmission from non-human primates (NHP) to humans.

Over the past few decades, the frequency of interaction between non-human primates (NHP) and humans has increased, breaching the natural boundaries between humans and animals and increasing the risks of zoonotic transmission and pandemics [1]. Humans most likely come into contact with NHP in certain low-income countries where wild animals are the main source of meat [2]. Additionally, in many countries, the hunting of NHP is a common practice, and the sale of wild meat is legal. The illegal trade and transport of large amounts of live NHP and meat in the United States and Europe has increased the risk of transmission of pathogens from NHP to humans [3]. Furthermore, the development of roads and agricultural projects have reduced wildlife habitats and led to changes in the ecosystems, resulting in increased human–NHP interactions [4]. NHPs are highly similar to humans in terms of tissue structure, immunity profile, physiology, and metabolism. The use of NHP as experimental models in biomedical research has increased, and people working in primate research centers are at a high risk of infection [1]. Moreover, the growth of international tourism is associated with a risk of pathogen spread. For instance, in Bali, more than 700,000 tourists visit the Monkey Temple each year. Furthermore, NHPs have transmitted pathogens to more than 33 ecotourists visiting the wild great apes in Africa [5]. Vectors containing viral pathogens can also be transmitted between NHP and humans, increasing the likelihood of epidemics (Figure 1) [1].

**Figure 2:**
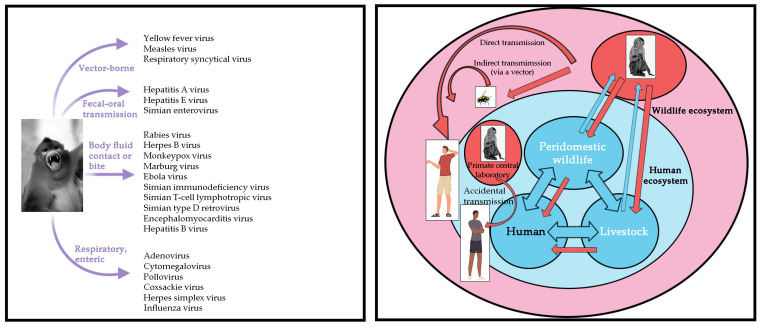
The schematic representation summarizes the known infectious viral pathogens reported to have been transmitted from NHP to humans. Left: NHP transmits viral pathogens to humans. Right: Diagram of interspecific transmission of the pathogen from NHP to humans.

**Figure 3:**
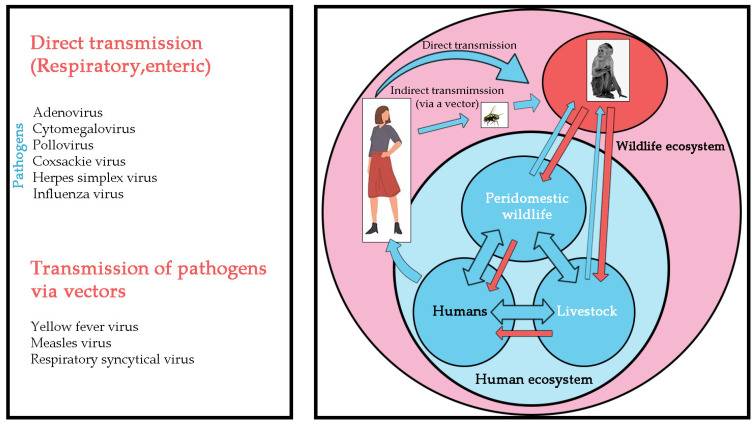
The schematic representation summarizes the known infectious viral pathogens reported to have been transmitted from humans to NHP. Left: Human diseases that threaten NHP. Right: Diagram of interspecific transmission of the pathogen from humans to NHP.

Global public health faces increasing challenges, as “new infectious diseases are emerging constantly, and old infectious diseases are emerging one after another” [6]. The COVID-19 pandemic has affected the world’s population and has not yet been effectively controlled. In addition, the World Health Organization announced mpox (or monkeypox) as a public health emergency of international concern in July 2022. In recent decades, deadly viruses such as rabies virus, herpes B virus, Marburg virus, Ebola virus, human immunodeficiency virus, and MPXV have had opportunity to transfer from NHP to humans by air droplets, fecal-oral contamination, cutaneous contact, bites, or arthropod vectors (Figure 2). Similarly, herpes simplex virus, influenza virus, and yellow fever virus can be transmitted from humans to NHP. The risk of infection is a direct consequence of the pathogen’s mode of transmission and its ability to survive in aerosols, soil, water, food, or feces (Figure 3). 

Given the diversity of these pathogens and their ability to infect hosts, bidirectional transmission may also occur [7]. Halting the transmission of viruses via the zoonotic route will eventually curb both human infection with these viruses and the resulting epidemics [8]. In both cases discussed above, the threat is well-understood and preventive measures can be implemented or treatment can be initiated immediately after an individual is infected. In contrast, unknown pathogens continue to pose risks to global health. Therefore, it is important to identify the pathogens carried by NHP that may spread to humans to prepare for possible epidemics and outbreaks.

New zoonotic and fatal pathogens with high epidemic potential and mortality have threatened global health security for centuries. Therefore, it is necessary to systematically understand infectious pathogens carried by NHP and their research status, diagnostic methods, prevention, and treatment measures to prevent outbreaks of human-infectious diseases (Table 1).

## 2. Monkeypox Virus (MPXV)

The MPXV is a zoonotic orthopoxvirus that causes mpox disease, which is similar to smallpox but with a milder rash and lower mortality. The presence of lymphadenopathy in MPXV is a striking clinical feature that distinguishes it from smallpox [21]. MPXV can be transmitted from monkeys and terrestrial rodents [22]. According to the first report in 1958, monkeys and other susceptible animals were infected with MPXV only in the tropical rain forests of Central and West Africa. Since then, mpox has spread from Middle and West Africa to North America and many outbreaks have been recorded worldwide [23]. Among the Old World NHP, *Macaca fascicularis*, *Cercocebus atys*, *Pongo pygmaeus*, and *Pan troglodytes* can be infected with MPXV, whereas among the New World NHP, *Callithrix jacchus* is susceptible to MPXV infection [24].

**Figure 4:**
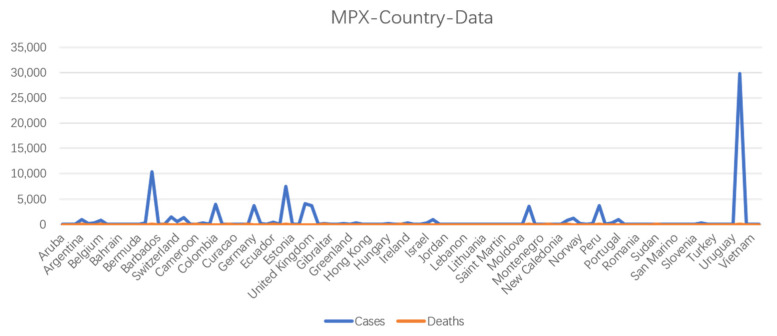
Number of cases and deaths caused MPXV infection, according to country, as of 28 December 2022.

**Figure 5:**
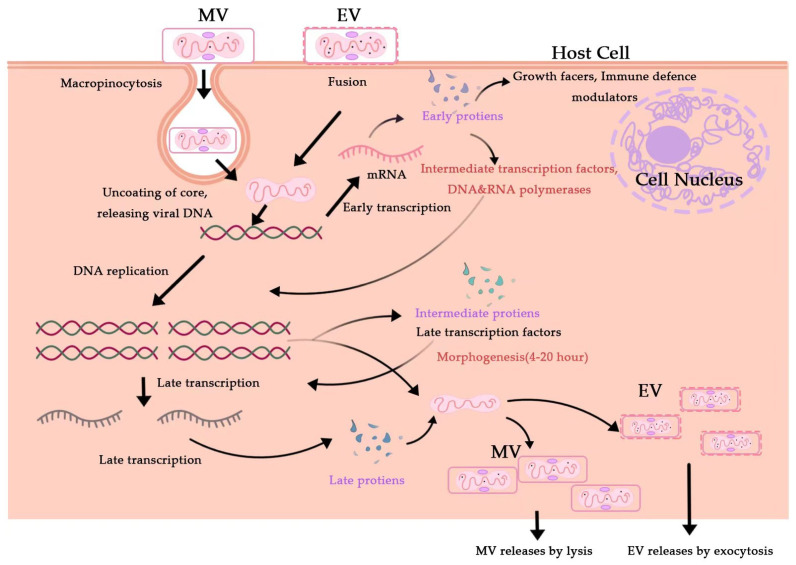
Cytosolic MPXV pathways for the viral life cycle. Enveloped Virion (EV) enters the host cell by fusion and the mature virion (MV) by micropinocytosis or fusion.

Since 2016, at least 1000 human infections with MPXV have been reported worldwide [25]. In May 2022, an outbreak of MPXV began in Europe and the United States, with a fatality rate of as high as 10% [3]. As of 28 December 2022, over 83,000 laboratory-confirmed human mpox cases were been reported in 110 countries worldwide (https://www.cdc.gov/) accessed on 6 January 2023 (Figure 4). Most cases are caused by human contact with ill monkeys or disease-susceptible animals, whereas some infections are caused by close-range droplet transmission from an infected person. Humans are affected by two distinct clades of this pathogenic disease, the West African and the Central African clades. The mpox cases identified in the United States are caused by two different MPXV strains, both of which belong to the West African clade. Some countries have initiated the implementation of methods to prevent and control mpox. Although some information on the transmission route (Figure 5) is available, clinical characteristics, epidemiology, and strategies for the diagnosis and treatment of mpox remain unclear [26].

Patients initially diagnosed with mpox typically have a temperature of above 38.5 °C and swollen lymph nodes [27]. The clinical manifestations of MPXV infection are difficult to distinguish from those of diseases caused by other poxviruses. Thus, a combination of methods based on clinical symptoms, epidemiological analyses, and laboratory testing is needed to accurately diagnose the disease. Infection must be confirmed with laboratory testing, as the clinical and epidemiological criteria may vary depending on the actual situation and geographical location [28]. Laboratory methods mainly include immunological and nucleic acid detection, including viral isolation and culture, base factor sequencing, polymerase chain reaction (PCR), and immunohistochemistry. Because these procedures involve the use of live viruses, they should be strictly performed in a certified class Ⅲ biosafety (BSL-3) laboratory (P3). Viral isolation and culture are standard methods for diagnosing viral diseases. MPXV is isolated from the viral samples and morphologically analyzed using electron microscopy. The herpetic fluid, pustules, or crusts are collected to prepare a suspension, and the morphological characteristics of the virions are observed by performing electron microscopy. The virions are brick-shaped or elliptic, 200 nm × 250 nm in size and contain cysts. As poxvirus species are morphologically indistinguishable, electron microscopy can only distinguish poxviruses from viruses of other genera but cannot confirm MPXV. Thus, molecular genetic methods such as PCR, real-time PCR, and next-generation sequencing must be used to confirm the presence of MPXV [29]. Genome-wide sequencing is the gold standard for differentiating MPXV from other orthopoxisome viruses [30]. Enzyme-linked immunosorbent assays can also be used to detect specific antibodies, namely IgM and IgG, in the serum of patients after 5 and 8 days of infection, respectively [31]. However, due to the conserved genome between MPXV and other poxviruses, extensive antigenic cross-reactions occur among poxviruses causing low specificity. Therefore, antibody reactions cannot accurately identify MPXV and are often used in epidemiological investigations [32]. In a new study, researchers identified the holoenzyme structure of MPXV DNA polymerase using cryogenic electron microscopy. They also found that the MPXV DNA polymerases bind in relatively similar ways to DNA polymerases found in the B family polymerases of other viral species, suggesting a degree of conservation in evolution that could help in the development of treatments and vaccines against MPXV viruses [33].

To date, no specific vaccine against mpox has been developed. MPXV is closely related to the smallpox virus, and the smallpox vaccine can provide both cross-immunity and strong protection against monkeypox [34], with a prevention rate of 85% or a reduction in disease severity [35]. It has been used for interconnectors in the United Kingdom [36]. No specific antiviral drug is available for mpox treatment. The main clinical treatment of mpox is symptomatic treatment and supportive therapy to reduce symptoms and control complications. Anti-variola drugs exhibit anti-MPXV properties and are promising treatment options for monkeypox. For patients with severe infection, antiviral therapy, typically with tecovirimat, cidofovir, or brincidofovir, is recommended [9].

Mpox is a global public health problem that affects not only African countries, such as Central and West African countries but also the entire world [10,37]. As the largest and most widespread mpox epidemic to date outside the African continent, the disease has gained the attention of international health organization [38]. Early diagnosis and avoiding close contact with infected people, animals, and potentially contaminated objects can help prevent MPXV infections [39]. To effectively reduce the transmission risk of MPXV, it is essential to strengthen disease prevention, surveillance, and international cooperation [40]. Further research on mpox as well as prevention and control measures should be conducted.

## 3. Rhesus Herpesviruses

Monkey B virus (MBV) belongs to the family Herpesviridae and the genus Herpes simplex virus in the Alpha-herpesviruses subfamily [41]. Rhesus macaques are the natural hosts of this virus, which is mainly transmitted through mating, scratches, or bites. Once infected, monkeys typically carry the virus for life and show few or no symptoms. These symptoms, including oral herpes-like lesions, such as oral ulcers, and conjunctivitis without symptoms of genital tract damage, are similar to those of human infection with Herpes simplex virus 1 and 2. These lesions eventually heal without treatment [42]. In 1932, Sabin et al. reported the first isolation of the virus from the brain and spleen of a patient suffering from encephalitis after being bitten by a healthy-looking rhesus monkey. Around 200 human exposures per month are documented by the National Institutes of Health, and many more undocumented exposures are likely, emphasizing the possibility of transmission [43]. On 16 July 2021, the Chinese Center for Disease Control and Prevention Weekly Report (English) documented the first human case of MBV infection in China. China had not reported any obvious or fatal cases of MBV infection previously [44]. MBV is a BSL-3 pathogen; thus, research on this virus must be performed in a BSL-3 laboratory. The disease can be initially diagnosed based on epidemiological data (such as contact history), clinical symptoms, laboratory tests, such as viral isolation and identification, and serological, histopathological, and molecular biological examinations. Currently, there is no effective vaccine or therapy against MBV. In 2014, the Chinese Center for Disease Control and Prevention published four scenarios in which humans were exposed to MBV for prophylactic antiviral therapy [45]. The infection rate of MBV in rhesus monkeys is high and leads to a fatality rate of 70–80%, with the positive rate of antibodies increasing with age. Therefore, single-cage breeding should be performed, along with regular quarantine and elimination of positive monkeys to gradually establish MBV-free monkeys. Wild monkeys should be considered as infected with MBV, strictly isolated, and quarantined, and those confirmed to be negative for MBV can be used for research. 

Simian cytomegalovirus (SCMV) belongs to the Herpesviridae family of beta-herpesvirus. Rhesus monkeys are the only host of SCMV, and humans and other animals are not susceptible to infection with this virus [11]. Although natural infection with SCMV is common in rhesus monkeys, infection in healthy animals is occult. Many of the pathological symptoms of rhesus monkeys infected with SCMV are similar to those of humans infected with human cytomegalovirus (HCMV). Thus, these infected monkeys can be used as an HCMV model to study the etiology, immunosuppression, and fetal malformation effects of SCMV [46]. Although progress in the research of the SCMV model has been achieved, there is no approved vaccine for HCMV or SCMV. Dennis et al. tested the following two vaccine strategies that may have synergistic effects in reducing the rate of horizontal SCMV transmission: (i) vaccination with recombinant glycoprotein B to interfere with mucosal SCMV acquisition and (ii) vaccination with recombinant viral interleukin-10 to reduce viral shedding [47]. Although most wild and captive rhesus macaques are SCMV-seropositive, the natural history of the viral acquisition remains unclear. Infant rhesus macaques typically become IgG-seropositive by one year of age, but it remains to be confirmed whether the virus was acquired from their mothers [13]. Permar et al. found that exposure to SCMV shed in breast milk and other maternal fluids postpartum results in less efficient mother-to-child transmission of SCMV compared to HCMV [48]. These researchers also determined the characteristics of the early maternal SCMV-specific humoral immune response in rhesus monkeys to primary SCMV infection; further studies are needed to identify antibody responses to serve as vaccine targets to eliminate congenital HCMV transmission [49]. The SCMV genome has been fully sequenced and annotated [50]. A comparison of the coding potential of the SCMV and HCMV genomes showed that the degree of conservation of viral open reading frames and gene families between SCMV and HCMV is higher than previously estimated and higher than that between Murine cytomegalovirus and HCMV [51]. Früh found that the natural killer cell receptor NKG2D plays an important role. In vivo, SCMV infects its hosts by intercepting NKG2DLs, which are highly conserved in rhesus monkeys. Interestingly, SCMV lacks the homologs UL16 and UL142 and instead uses the homologs UL148 and Rh159 to prevent NKG2DL surface expression [52]. The diagnosis of SCMV infection relies on laboratory tests, including virus isolation and culture, serum antibody testing, antigen testing, and viral nucleic acid detection. SCMV is an opportunistic infectious agent that infects an immunocompromised host. Active infection can cause severe rhesus cytomegalovirus disease. Immunocompetent hosts do not exhibit clinical symptoms after infection with SCMV and do not require treatment. There is currently no effective treatment for systemically disseminated infections in animals with low immunity, and symptomatic treatment and supportive therapy can be applied when necessary [53].

## 4. Simian Retroviruses

Simian immunodeficiency virus (SIV) can cause immunodeficiency in macaques. African primates are the natural hosts of SIV. Experimental infection of non-natural hosts, such as rhesus monkeys, led to the suppression of the host immune responses, subsequently resulting in simian-acquired immunodeficiency syndrome [54]. SIV is a BSL-3 pathogen, and related research must be performed in a BSL-2/3 laboratory (BSL-3 operating procedures in a BSL-2 facility). In addition to using epidemiological data and clinical examination, the infection is diagnosed through laboratory virus isolation, as well as serological, histopathological, and molecular biological tests. The characteristics of SIV infection in rhesus monkeys are similar to those of human immunodeficiency virus-1 infection. Thus, rhesus monkeys are considered the “gold standard” model for studying simian-acquired immunodeficiency syndrome. Additionally, research on SIV has advanced. David et al. found that most SIVs use Nef protein, which has numerous functions, including downregulation of CD3, CD4, and major histocompatibility complex class I; enhancing viral infectivity; and downregulating simian tetherin to overcome the immune responses. He et al. found that SIV and human immunodeficiency virus-1 exhibit similar molecular patterns, resulting in resistance to various inhibitors of peptide- and lipopeptide-based membrane fusion [14]. There is currently no safe and effective vaccine for controlling the spread of SIV in wild African monkeys [55]. Therefore, measures, such as isolation, strict control, and elimination of infected monkeys, must be implemented for monkeys introduced from African countries and other epidemic areas to prevent the spread of SIV infection. Because of the potential of SIV to infect humans, laboratory workers and farm workers who may come into contact with infected animal blood, tissues, cultures, and infected equipment should use personal protective clothing and equipment.

Simian T-cell leukemia virus type 1 (STLV-1) was first discovered in Japanese macaques in 1982 and classified as belonging to the *Del-taretrovirus* genus [56]. According to the provirus nucleic acid sequence and serological response, STLV was subdivided into STLV-1, STLV-2, STLV-3, and STLV-4. STLV is associated with T-lymphoid leukemia and lymphoid neoplasms in NHP. In most cases, this virus has a long incubation period and infected NHP do not exhibit clinical symptoms for many years [57]. Notably, the prevalence of STLV-1 infection in Japanese macaques is approximately >60%, which is much higher than in other NHPs. STLV-1 has been detected in more than 30 Old World sub-Saharan primates, including monkeys and apes in Africa and Asia, whereas STLV-3 has been recorded in only a few African monkeys [58]. In contrast, the distribution of STLV-2 and STLV-4 appears to be much narrower, as determined by the extensive screening of different primates throughout West and Central Africa. STLV-4 has only been detected in western lowland gorillas in Cameroon, whereas STLV-2 appears to be restricted to captive bonobos (*Pan paniscus*) housed in zoos in primate centers in Europe and the United States. NHP is also a natural host for STLV-I [59]. However, the mechanism and route of transmission of STLV-1 remain unknown. Afonso et al. isolated the STLV-1 virus-Mra18C9 strain from Indian monkeys (*Macaca radiata*), and then determined the complete sequences and performed phylogenetic analysis. Interestingly, Mra18C9 lacks three auxiliary open reading frames; therefore, to determine whether deletion of the auxiliary protein is specific to this strain, the complete STLV-1 genome available from GenBank was analyzed. The results showed STLV-1 commonly lacks one or multiple secondary open reading frames [60]. Akari et al. suggested that frequent horizontal and mother-to-child transmission contributes to the high prevalence of STLV-1 infection in Japanese macaques [59]. Co-infection with STLV-1 is associated with an increased proviral load of simian foamy virus (SFV) in the peripheral blood of NHP naturally infected with SFV and STLV-1 [61]. STLV infection can be diagnosed using two types of methods: viral nucleic acid detection and antibody detection. Among them, the PCR method to detect viral nucleic acid provides the most direct indication of STLV infection [62]. A commercial STLV-1 enzyme-linked immunosorbent assay antibody detection kit was developed in the United States [63]. Epidemiological investigations showed that STLV-1 infection exists in Asian NHP. Asia is one of the world’s richest regions for NHP and the primary supply of experimental monkeys; therefore, it is important to detect and prevent STLV-1 in this region. Compared with other NHP viruses, the infection rate and prevalence of STLV are relatively low, which is beneficial for controlling disease spread. However, STLV has not yet been widely examined.

Simian type D retrovirus (SRV) is an enveloped, single-stranded RNA virus belonging to the genus *Beta-retrovirus*. Depending on the host species, virus, sex, and various environmental factors, the symptoms of infection may be absent, mild, or even severe enough to produce fatal simian-acquired immunodeficiency syndrome [15]. Owing to the different stages of viral infections and varying host-immune responses, it is necessary to use a combination of laboratory diagnoses including serological and viral molecular biology techniques to detect the viruses. Vaccines against inactivated SRV type Ⅰ and outer membrane glycoprotein vaccines expressing simian SRV types Ⅰ, II, and III in vaccinia virus vectors have been developed. However, it is unknown whether these vaccines can provide cross-protection between simian retrovirus types I–V. Additionally, further analyses are required to determine whether vaccines made with vaccinia virus vectors will interfere with research data [16]. Two cases of human infection with SRV, which may have been due to long-term exposure to macaques, have been reported. Therefore, breeding managers who have direct contact with macaques or laboratory workers who may be exposed to the virus should adhere to a minimum of BSL-2 protection measures to prevent direct exposure to the virus [64]. A novel endogenous retrovirus discovered in sifaka (*Propithecus coquereli*) was reported in 2021. The complete genome sequence of the retrovirus was identified, characterized, and named as prosimian SRV type Ⅰ [17]. Additionally, Grant et al. developed a diagnostic method for distinguishing endogenous and exogenous SRV [65]. Zhu et al. demonstrated that infection with SRV-8, a newly isolated SRV subtype, triggers autophagy and apoptotic pathways in Jurkat T lymphocytes [66]. Togami et al. found that zidovudine and tenofovir disoproxil fumarate, in sub-micromolar to nanomolar dosages, potently inhibit SRV-4 infection; conversely, non-nucleoside reverse transcriptase inhibitors and protease inhibitors showed no activity against SRV-4 [67]. Human infection with SRV has no clear clinical manifestations [68]; however, to prevent virus transmission, PCR is performed and model animals are regularly tested for viral antibodies, and animals that test positive for the virus are immediately removed.

SFV is also a simian retrovirus and has been reported in 1–4% of people working with NHP in zoos, primate centers, and laboratories and in approximately 24% of staff who have been bitten or scratched by gorillas or chimpanzees [69]. SFV co-evolved with Old World primates over 3–100 million years ago, adapted well to these hosts, and is considered non-pathogenic [70]. SFV is readily transmitted between individuals via saliva, and several reports suggest that the active replication of SFV is largely restricted to the epithelial cells of the oral mucosa in NHP [71]. However, viral DNA has also been detected in CD4^+^ and CD8^+^ T cells, as well as in B cells in vivo, and it was previously thought that peripheral blood mononuclear cells are the sites of latency of foamy viruses [72].

## 5. Ebola Virus (EBOV)

EBOV infection can lead to Ebola hemorrhagic fever, which is one of the most virulent zoonotic infectious diseases, with a mortality rate of as high as 90% [73]. In October 1989, 100 rhesus monkeys transported from the Philippines to the United States suddenly became ill and more than 60 died. The analysis confirmed that the monkeys were infected with EBOV, creating large concerns. The WHO classified EBOV as the most serious BSL-4 virus that can affect humans and relevant experimental procedures must be conducted in a highly secure BSL-4 laboratory [74]. The symptoms of EBOV infection are similar to those of Marburg hemorrhagic fever. According to the WHO, the death toll of EBOV infection is continuously increasing; to date, 1145 deaths among 2127 infected persons have been reported. Persistent EBOV infection in rhesus monkeys can serve as a model for persistent EBOV infection in humans, which can be used to study the sequelae of EBOV disease and develop therapies to eliminate EBOV persistence [75]. Although NHPs are a source of EBOV infection in humans, EBOV did not originate from NHP. Like humans, monkeys are infected through direct contact with a natural medium or via a chain of transmission. EBOV infection is diagnosed based on epidemiological data, clinical manifestations, positive laboratory tests for the viral antigen, positive serum-specific IgM antibody, and other actors. Serological tests include indirect immunofluorescence assay, enzyme-linked immunosorbent assay, radioimmunoassay, plaque reduction, and neutralization tests. Molecular biological tests generally involve nucleic acid amplification methods, such as reverse transcription PCR. Three vaccines and two antibody-based vaccine therapeutics have been approved in recent years for EBOV disease prevention [76].

## 6. Simian Hemorrhagic Fever Virus (SHFV)

SHFV is a severe and highly lethal infectious disease that can infect captive macaques. The disease is only transmitted among macaques and is characterized by hemorrhage and fever. Other species of monkeys are chronically infected with the virus but do not develop the disease. The clinical signs and disease course closely resemble those of other hemorrhagic fever virus infections, such as Marburg, Ebola Zaire, and Lassa virus infection in macaques [77]. The RNA virus gene consists of 15,000 nucleotides. The disease occurs in primates in the United States, Britain, Russia, and other countries [12]. The source of infection may be monkeys infected by the virus or those with an occult infection; however, the serological tests cannot determine whether wild macaques and European, Asian, and African monkeys can also be infected with the virus. Clinical manifestations include extensive bleeding, high fever, and mortality. The virus has also been isolated from wild red uranium monkeys in Uganda. Therefore, wild primates are natural reservoirs of the virus [78].

SHFV causes lethal hemorrhagic fever and induces a strong inflammatory response in macaques but causes asymptomatic persistent infection in baboons. Margo et al. isolated SHFV, which induces hemorrhagic fever in Japanese macaques, from persistently infected baboons [79]. In addition, Tony et al. found abnormal SHFV diversity in African wild primate communities [80]. Vatter et al. showed that the cell entry of SHFV is dependent on CD163 and a reticulin-mediated endocytosis-like pathway [81]. Jens et al. found that SHF is a disease of rhesus monkeys likely caused by numerous different simian arterioviruses [82]. SHFV invades cells via low pH-dependent, actin-independent endocytosis, presumably with the help of cell surface proteins. The virus can infect humans; therefore, monkeys have been used as a research model for human hemorrhagic fever disease. Johnson et al. compared experimental models following SHFV infection in patas and monkeys to evaluate the mechanisms of the host immune response associated with viral hemorrhagic fever and pan-viral hemorrhagic fever countermeasures [83].

SHFV can be clinically diagnosed based on characteristic clinical symptoms, pathological changes, and epidemiological characteristics combined with serological, histopathological, and molecular biological tests. The feeding and management of monkeys and environmental disinfection should be strengthened to prevent mechanical transmission by personnel, equipment, rodents, insects, and other vectors. Currently, there are no specific and effective vaccines and treatments for SHFV infection [18].

## 7. Simian Adenovirus (SADV)

SADV can produce various symptoms, including pneumonia, pharyngitis, gastroenteritis, and conjunctivitis. It is mainly transmitted via the fecal–oral route and primarily infects primates, such as macaques, vervet monkeys, African green monkeys, chimpanzees, and baboons [84]. Adenovirus is a common virus in the respiratory and digestive tracts of monkeys. Some scholars have suggested that infection by SADV has strict species specificity, and there are reports of potential cross-transmission between NHP species [19]. SADV and human adenovirus are closely related and share a common soluble complement-binding antigen. Some strains of SADV can cause respiratory diseases, such as pneumonia in juvenile monkeys. The clinical symptoms caused by the infection are similar, making it an ideal model for human adenovirus research [85]. SADV can infect various types of monkeys and has a high mortality rate; thus, experimental monkeys should be kept in single cages and quarantined regularly, and monkeys that test positive should be eliminated to gradually establish adenovirus-free monkeys [86]. When analyzing the status of SADV infection in animals and detecting SADV contamination of biological products, the combined use of various detection methods, such as pathogen isolation, PCR, transmission electron microscopy, immunofluorescence detection, and nucleic acid hybridization, is important for comprehensively evaluating SADV. The establishment of rapid, sensitive, simple, and accurate SADV detection methods, will enable the discovery of new SADV strains. There is currently no effective therapeutic drug, and SADV is often used as a recombinant vaccine carrier [87].

## 8. Simian Parvovirus (SPV)

SPV is a single-stranded DNA virus recently identified in cynomolgus monkeys with severe anemia [88], although the infection it causes is typically asymptomatic or only manifests as a mild febrile illness.

Under normal circumstances, animals mostly have a latent infection without corresponding clinical symptoms and do not require treatment. However, blood transfusions can be performed for patients with clinically severe anemia or in experimental animals with severe anemia due to immunosuppressive therapy. General treatment and supportive therapy such as fluid replacement and antiviral methods can be used to treat the infection. SPV is an important pathogen in surgically manipulated cynomolgus monkeys, particularly in those that are immunocompromised. Clinically asymptomatic SPV infections are readily transmitted in the environment once colonies are introduced, and the latent virus can be transmitted through organs [89]. SPV can infect human bone marrow monocytes in vitro and in vivo and should be considered a potential zoonotic disease [90]. SPV is clinically diagnosed based on characteristic clinical symptoms and epidemiological characteristics combined with serological, hemagglutination, hemagglutination inhibition, histopathological, and molecular biological tests. No vaccines or drugs for prophylaxis in primates have been reported to date [91]. Timely and effective isolation of ill primates and virus-carrying animals to establish SPV-negative animal populations is fundamental to preventing the disease [20].

## 9. Conclusions

Due to the emergence of acquired immunodeficiency syndrome as well as the recent outbreak of monkeypox, it is imperative to understand the risks of the potential transmission and spread of emerging infectious diseases from NHP to humans. In case of unclear threats, it is necessary to determine which behaviors increase exposure, the types of pathogens that have crossed the species barrier, the importance of this risk to health following infection or frequent contact with NHP, and the appropriate measures. To this end, the habitats of NHP should be protected, and policies and regulations should be issued to restrict the consumption and trading of meat from NHP and to strengthen the inspection and quarantine of experimental animals. Furthermore, self-protection must be emphasized for susceptible animal husbandry managers, tourists, clinical veterinarians, and researchers. Additionally, public health professionals, national authorities, and the media should more readily divulge information to the general public to raise awareness. Active monitoring and health assessments of NHP populations are also necessary. 

The diagnosis of NHP viruses relies on the combined use of multiple detection methods; there are no effective vaccines or specific therapeutic drugs for most NHP viruses. The pathogens are not well-understood, and the existing data are limited and outdated. To effectively reduce the risk of zoonosis transmission, it is essential to strengthen prevention, monitoring, and international cooperation. Additionally, research and the development of specific vaccines and drugs should be strengthened. Moreover, further studies are urgently needed to determine the mechanisms of replication, infection, and transmission of pathogens of infectious diseases in NHP to prevent outbreaks in humans. In summary, our review of the updated research evidence, diagnostic methods, prevention, and treatment measures, may be useful for limiting the spread of several common viral pathogens by NHP and providing ideas for preventing zoonotic diseases with epidemic potential.

## Figures and Tables

**Table 1:** Symptoms, diagnostic methods, prevention, and treatment measures to limit the spread of several common non-human primates (NHP) viral pathogens.

Pathogens	Symptoms	Diagnostic Methods	Vaccination	Antiviral Drugs	Reference
Monkeypox virus	Milder rash and lower mortality	RT-PCR, NGS, electron microscope, Immunological	No effective measures	No effective curative treatment	[9,10]
Monkey B virus	Oral herpes-like lesions, such as oral ulcers, conjunctivitis	RT-PCR, NGS, Immunological, Histopathological	No effective measures	Effective suppressive treatment but not curative	[11]
Ebola virus; Marburg virus	Severe hemorrhagic fever fatal cases	Epidemiological data, clinical manifestations, positive laboratory tests for viral antigen and positive serum	No effective measures	mAbs therapy, Inmazeb, Ebanga	[12]
Simian cytomegalovirus	Basically recessive and does not show any clinical symptoms and signs	virus isolation and culture, serum antibody testing, antigen testing, and viral nucleic acid molecular biology testing	No effective measures	No effective curative treatment	[13]
Simian immunodeficiency virus	Monkey-acquired immunodeficiency (SAIDS)	Laboratory virus isolation, serological tests, histopathological diagnosis, and molecular biological to diagnosis	No effective measures	No effective curative treatment	[14]
Simian type D retrovirus	SAIDS	Serology and viral molecular biology	Inactivated SRV-Ⅰ, outer membrane glycoprotein vaccines expressing simian retrovirus	AZT and TDF, potently inhibited SRV-4 infection	[15,16,17]
Simian hemorrhagic fever virus	Hemorrhage and fever	Clinical symptoms, pathological, epidemic characteristics, serological tests, molecular biological diagnosis	No effective measures	No effective curative treatment	[18]
Simian adenovirus	Pneumonia, pharyngitis, gastroenteritis, conjunctivitis	Pathogen isolation, PCR, transmission electron microscopy, immunofluorescence detection, nucleic acid hybridization	No effective measures	No effective treatment	[19]
Simian parvovirus	Not apparent or causes only mild febrile illness	Clinical symptoms, epidemic characteristics, serological tests, hemagglutination and hemagglutination inhibition, histopathological and molecular biological	No effective measures	No effective curative treatment	[20]

Abbreviations: AZT, zidovudine; NGS, next-generation sequencing; mAb, monoclonal antibody; RT-PCR, reverse transcription polymerase chain reaction; SRV-4, simian retrovirus type 4; TDF, tenofovir disoproxil fumarate.

## Data Availability

Not applicable.

## References

[1] Devaux C.A., Mediannikov O., Medkour H., Raoult D. (2019). Infectious Disease Risk Across the Growing Human-Non Human Primate Interface: A Review of the Evidence. Front. Public Health.

[2] Brashares J.S., Golden C.D., Weinbaum K.Z., Barrett C.B., Okello G.V. (2011). Economic and geographic drivers of wildlife consumption in rural Africa. Proc. Natl. Acad. Sci. USA.

[3] Shanee N., Mendoza A.P., Shanee S. (2017). Diagnostic overview of the illegal trade in primates and law enforcement in Peru. Am. J. Primatol..

[4] Estrada A., Garber P.A., Rylands A.B., Roos C., Fernandez-Duque E., Di Fiore A., Nekaris K.A., Nijman V., Heymann E.W., Lambert J.E. (2017). Impending extinction crisis of the world’s primates: Why primates matter. Sci. Adv..

[5] Kabuga A.I., El Zowalaty M.E. (2019). A review of the monkeypox virus and a recent outbreak of skin rash disease in Nigeria. J. Med. Virol..

[6] Espinosa S., Branch L.C., Cueva R. (2014). Road development and the geography of hunting by an Amazonian indigenous group: Consequences for wildlife conservation. PLoS ONE.

[7] Dunay E., Apakupakul K., Leard S., Palmer J.L., Deem S.L. (2018). Pathogen Transmission from Humans to Great Apes is a Growing Threat to Primate Conservation. Ecohealth.

[8] Lai C.C., Hsu C.K., Yen M.Y., Lee P.I., Ko W.C., Hsueh P.R. (2022). Monkeypox: An emerging global threat during the COVID-19 pandemic. J. Microbiol. Immunol. Infect..

[9] Cohen-Gihon I., Israeli O., Shifman O., Erez N., Melamed S., Paran N., Beth-Din A., Zvi A. (2020). Identification and Whole-Genome Sequencing of a Monkeypox Virus Strain Isolated in Israel. Microbiol. Resour. Announc..

[10] Ferdous J., Barek M.A., Hossen M.S., Bhowmik K.K., Islam M.S. (2023). A review on monkeypox virus outbreak: New challenge for world. Health Sci. Rep..

[11] Weigler B.J. (1992). Biology of B virus in macaque and human hosts: A review. Clin. Infect. Dis..

[12] Bennett R.S., Logue J., Liu D.X., Reeder R.J., Janosko K.B., Perry D.L., Cooper T.K., Byrum R., Ragland D., St Claire M. (2020). Kikwit Ebola Virus Disease Progression in the Rhesus Monkey Animal Model. Viruses.

[13] Bowman J.J., Burbelo P.D., Gill R.B., Sauri M.A., Schmitt J.M., Cohen J.I. (2014). A seroprevalence study of primate workers for asymptomatic rhesus cytomegalovirus infection. J. Clin. Virol..

[14] Burwitz B.J., Malouli D., Bimber B.N., Reed J.S., Ventura A.B., Hancock M.H., Uebelhoer L.S., Bhusari A., Hammond K.B., Espinosa Trethewy R.G. (2016). Cross-Species Rhesus Cytomegalovirus Infection of Cynomolgus Macaques. PLoS Pathog..

[15] Traina-Dorge V.L., Lorino R., Gormus B.J., Metzger M., Telfer P., Richardson D., Robertson D.L., Marx P.A., Apetrei C. (2005). Molecular epidemiology of simian T-cell lymphotropic virus type 1 in wild and captive sooty mangabeys. J. Virol..

[16] Montiel N.A. (2010). An updated review of simian betaretrovirus (SRV) in macaque hosts. J. Med. Primatol..

[17] Gardner M.B., Luciw P.A., Sawai E.T., Marthas M.L., Miller C.J., McChesney M.B., Lerche N.W., Pedersen N.C. (1996). Simian retrovirus vaccines: Simian retrovirus and simian immunodeficiency lentivirus. AIDS Res. Hum. Retrovir..

[18] Cai Y., Postnikova E.N., Bernbaum J.G., Yu S.Q., Mazur S., Deiuliis N.M., Radoshitzky S.R., Lackemeyer M.G., McCluskey A., Robinson P.J. (2015). Simian hemorrhagic fever virus cell entry is dependent on CD163 and uses a clathrin-mediated endocytosis-like pathway. J. Virol..

[19] Cornish J.P., Moore I.N., Perry D.L., Lara A., Minai M., Promeneur D., Hagen K.R., Virtaneva K., Paneru M., Buechler C.R. (2019). Clinical Characterization of Host Response to Simian Hemorrhagic Fever Virus Infection in Permissive and Refractory Hosts: A Model for Determining Mechanisms of VHF Pathogenesis. Viruses.

[20] Brown K.E., Liu Z., Gallinella G., Wong S., Mills I.P., O’Sullivan M.G. (2004). Simian parvovirus infection: A potential zoonosis. J. Infect Dis..

[21] Isidro J., Borges V., Pinto M., Sobral D., Santos J.D., Nunes A., Mixao V., Ferreira R., Santos D., Duarte S. (2022). Phylogenomic characterization and signs of microevolution in the 2022 multi-country outbreak of monkeypox virus. Nat. Med..

[22] Epstein J.H., Price J.T. (2009). The significant but understudied impact of pathogen transmission from humans to animals. Mt. Sinai J. Med..

[23] Reynolds M.G., Doty J.B., McCollum A.M., Olson V.A., Nakazawa Y. (2019). Monkeypox re-emergence in Africa: A call to expand the concept and practice of One Health. Expert Rev. Anti Infect. Ther..

[24] Durski K.N., McCollum A.M., Nakazawa Y., Petersen B.W., Reynolds M.G., Briand S., Djingarey M.H., Olson V., Damon I.K., Khalakdina A. (2018). Emergence of Monkeypox—West and Central Africa, 1970–2017. MMWR Morb. Mortal. Wkly. Rep..

[25] McCollum A.M., Damon I.K. (2014). Human monkeypox. Clin. Infect. Dis..

[26] Sklenovska N., Van Ranst M. (2018). Emergence of Monkeypox as the Most Important Orthopoxvirus Infection in Humans. Front. Public Health.

[27] Fine P.E., Jezek Z., Grab B., Dixon H. (1988). The transmission potential of monkeypox virus in human populations. Int. J. Epidemiol..

[28] Heymann D.L., Szczeniowski M., Esteves K. (1998). Re-emergence of monkeypox in Africa: A review of the past six years. Br. Med. Bull..

[29] Doty J.B., Malekani J.M., Kalemba L.N., Stanley W.T., Monroe B.P., Nakazawa Y.U., Mauldin M.R., Bakambana T.L., Liyandja Dja Liyandja T., Braden Z.H. (2017). Assessing Monkeypox Virus Prevalence in Small Mammals at the Human-Animal Interface in the Democratic Republic of the Congo. Viruses.

[30] Alakunle E., Moens U., Nchinda G., Okeke M.I. (2020). Monkeypox Virus in Nigeria: Infection Biology, Epidemiology, and Evolution. Viruses.

[31] Silva N.I.O., de Oliveira J.S., Kroon E.G., Trindade G.S., Drumond B.P. (2020). Here, There, and Everywhere: The Wide Host Range and Geographic Distribution of Zoonotic Orthopoxviruses. Viruses.

[32] Bhattacharya M., Dhama K., Chakraborty C. (2022). Recently spreading human monkeypox virus infection and its transmission during COVID-19 pandemic period: A travelers’ prospective. Travel Med. Infect. Dis..

[33] Peng Q., Xie Y., Kuai L., Wang H., Qi J., Gao G.F., Shi Y. (2023). Structure of monkeypox virus DNA polymerase holoenzyme. Science.

[34] Moore M.J., Rathish B., Zahra F. (2022). Monkeypox. StatPearls.

[35] Osadebe L., Hughes C.M., Shongo Lushima R., Kabamba J., Nguete B., Malekani J., Pukuta E., Karhemere S., Muyembe Tamfum J.J., Wemakoy Okitolonda E. (2017). Enhancing case definitions for surveillance of human monkeypox in the Democratic Republic of Congo. PLoS Negl. Trop. Dis..

[36] Radonic A., Metzger S., Dabrowski P.W., Couacy-Hymann E., Schuenadel L., Kurth A., Matz-Rensing K., Boesch C., Leendertz F.H., Nitsche A. (2014). Fatal monkeypox in wild-living sooty mangabey, Cote d’Ivoire, 2012. Emerg. Infect. Dis..

[37] Yang Z. (2022). Monkeypox: A potential global threat?. J. Med. Virol..

[38] Brown K., Leggat P.A. (2016). Human Monkeypox: Current State of Knowledge and Implications for the Future. Trop. Med. Infect. Dis..

[39] Petersen B.W., Kabamba J., McCollum A.M., Lushima R.S., Wemakoy E.O., Muyembe Tamfum J.J., Nguete B., Hughes C.M., Monroe B.P., Reynolds M.G. (2019). Vaccinating against monkeypox in the Democratic Republic of the Congo. Antivir. Res..

[40] Adalja A., Inglesby T. (2022). A Novel International Monkeypox Outbreak. Ann. Intern. Med..

[41] Yinka-Ogunleye A., Aruna O., Dalhat M., Ogoina D., McCollum A., Disu Y., Mamadu I., Akinpelu A., Ahmad A., Burga J. (2019). Outbreak of human monkeypox in Nigeria in 2017-18: A clinical and epidemiological report. Lancet Infect. Dis..

[42] Cabanillas B., Valdelvira R., Akdis C.A. (2022). Monkeypox outbreak in Europe, UK, North America, and Australia: A changing trend of a zoonotic disease. Allergy.

[43] Gong Q., Wang C., Chuai X., Chiu S. (2022). Monkeypox virus: A re-emergent threat to humans. Virol. Sin..

[44] Eberle R., Jones-Engel L. (2018). Questioning the Extreme Neurovirulence of Monkey B Virus (*Macacine alphaherpesvirus* 1). Adv. Virol..

[45] Maxwell L.K., Black D.H., Wright G.E., Breshears M.A., Eberle R. (2020). Effective Prophylactic Therapy for Exposure to Monkey B Virus (*Macacine alphaherpesvirus* 1). Comp. Med..

[46] Johnston W.F., Yeh J., Nierenberg R., Procopio G. (2015). Exposure to Macaque Monkey Bite. J. Emerg. Med..

[47] Wang W., Qi W., Liu J., Du H., Zhao L., Zheng Y., Wang G., Pan Y., Huang B., Feng Z. (2021). First Human Infection Case of Monkey B Virus Identified in China, 2021. China CDC Wkly..

[48] Fan Q., Nelson C.S., Bialas K.M., Chiuppesi F., Amos J., Gurley T.C., Marshall D.J., Eudailey J., Heimsath H., Himes J. (2017). Plasmablast Response to Primary Rhesus Cytomegalovirus (CMV) Infection in a Monkey Model of Congenital CMV Transmission. Clin. Vaccine Immunol..

[49] Deere J.D., Chang W.L.W., Villalobos A., Schmidt K.A., Deshpande A., Castillo L.D., Fike J., Walter M.R., Barry P.A., Hartigan-O’Connor D.J. (2019). Neutralization of rhesus cytomegalovirus IL-10 reduces horizontal transmission and alters long-term immunity. Proc. Natl. Acad. Sci. USA.

[50] Abel K., Martinez J., Yue Y., Lacey S.F., Wang Z., Strelow L., Dasgupta A., Li Z., Schmidt K.A., Oxford K.L. (2011). Vaccine-induced control of viral shedding following rhesus cytomegalovirus challenge in rhesus macaques. J. Virol..

[51] Kaur A., Itell H.L., Ehlinger E.P., Varner V., Gantt S., Permar S.R. (2018). Natural history of postnatal rhesus cytomegalovirus shedding by dams and acquisition by infant rhesus monkeys. PLoS ONE.

[52] Truitt L.L., Yang D., Espinoza D.A., Fan X., Ram D.R., Mostrom M.J., Tran D., Sprehe L.M., Reeves R.K., Donahue R.E. (2019). Impact of CMV Infection on Natural Killer Cell Clonal Repertoire in CMV-Naive Rhesus Macaques. Front. Immunol..

[53] Hansen S.G., Strelow L.I., Franchi D.C., Anders D.G., Wong S.W. (2003). Complete sequence and genomic analysis of rhesus cytomegalovirus. J. Virol..

[54] Malouli D., Nakayasu E.S., Viswanathan K., Camp D.G., Chang W.L., Barry P.A., Smith R.D., Fruh K. (2012). Reevaluation of the coding potential and proteomic analysis of the BAC-derived rhesus cytomegalovirus strain 68-1. J. Virol..

[55] Norley S., Kurth R. (2004). The role of the immune response during SIVagm infection of the African green monkey natural host. Front. Biosci..

[56] Ziani W., Shao J., Wang X., Russell-Lodrigue K., Liu Y.Z., Montaner L.J., Veazey R.S., Xu H. (2021). Increased Proviral DNA in Circulating Cells Correlates with Plasma Viral Rebound in Simian Immunodeficiency Virus-Infected Rhesus Macaques after Antiretroviral Therapy Interruption. J. Virol..

[57] Poiesz B.J., Ruscetti F.W., Gazdar A.F., Bunn P.A., Minna J.D., Gallo R.C. (1980). Detection and isolation of type C retrovirus particles from fresh and cultured lymphocytes of a patient with cutaneous T-cell lymphoma. Proc. Natl. Acad. Sci. USA.

[58] Murata M., Yasunaga J.I., Washizaki A., Seki Y., Kuramitsu M., Tan W.K., Hu A., Okuma K., Hamaguchi I., Mizukami T. (2020). Frequent horizontal and mother-to-child transmission may contribute to high prevalence of STLV-1 infection in Japanese macaques. Retrovirology.

[59] Castro I., Giret T.M., Magnani D.M., Maxwell H.S., Umland O., Perry J.K., Pecotte J.K., Brasky K.M., Barber G.N., Desrosiers R.C. (2016). Cellular Immune Responses against Simian T-Lymphotropic Virus Type 1 Target Tax in Infected Baboons. J. Virol..

[60] Termini J.M., Magnani D.M., Maxwell H.S., Lauer W., Castro I., Pecotte J., Barber G.N., Watkins D.I., Desrosiers R.C. (2017). Simian T Lymphotropic Virus 1 Infection of Papio anubis: Tax Sequence Heterogeneity and T Cell Recognition. J. Virol..

[61] Afonso P.V., Fagrouch Z., Deijs M., Niphuis H., Bogers W., Gessain A., van der Hoek L., Verschoor E.J. (2019). Absence of accessory genes in a divergent simian T-lymphotropic virus type 1 isolated from a bonnet macaque (Macaca radiata). PLoS Negl. Trop. Dis..

[62] Alais S., Pasquier A., Jegado B., Journo C., Rua R., Gessain A., Tobaly-Tapiero J., Lacoste R., Turpin J., Mahieux R. (2018). STLV-1 co-infection is correlated with an increased SFV proviral load in the peripheral blood of SFV/STLV-1 naturally infected non-human primates. PLoS Negl. Trop. Dis..

[63] Liegeois F., Boue V., Mouacha F., Butel C., Ondo B.M., Pourrut X., Leroy E., Peeters M., Rouet F. (2012). New STLV-3 strains and a divergent SIVmus strain identified in non-human primate bushmeat in Gabon. Retrovirology.

[64] Anderson D.E., Torres J.V. (1999). Simian retrovirus receptor and neutralization mechanism by antibodies to the envelope glycoprotein. Viral Immunol..

[65] Sotir M., Switzer W., Schable C., Schmitt J., Vitek C., Khabbaz R.F. (1997). Risk of occupational exposure to potentially infectious nonhuman primate materials and to simian immunodeficiency virus. J. Med. Primatol..

[66] Apakupakul K., Deem S.L., Maqsood R., Sithiyopasakul P., Wang D., Lim E.S. (2021). Endogenization of a Prosimian Retrovirus during Lemur Evolution. Viruses.

[67] Grant R., Keele B., Kuller L., Watanabe R., Perret A., Smedley J. (2017). Identification of novel simian endogenous retroviruses that are indistinguishable from simian retrovirus (SRV) on current SRV diagnostic assays. J. Med. Primatol..

[68] Zhu J., Yang L., Zhang Q., Meng J., Lu Z.L., Rong R. (2020). Autophagy Induced by Simian Retrovirus Infection Controls Viral Replication and Apoptosis of Jurkat T Lymphocytes. Viruses.

[69] Togami H., Shimura K., Okamoto M., Yoshikawa R., Miyazawa T., Matsuoka M. (2013). Comprehensive in vitro analysis of simian retrovirus type 4 susceptibility to antiretroviral agents. J. Virol..

[70] Lerche N.W., Heneine W., Kaplan J.E., Spira T., Yee J.L., Khabbaz R.F. (1995). An expanded search for human infection with simian type D retrovirus. AIDS Res. Hum. Retrovir..

[71] Sandstrom P.A., Phan K.O., Switzer W.M., Fredeking T., Chapman L., Heneine W., Folks T.M. (2000). Simian foamy virus infection among zoo keepers. Lancet.

[72] Lambert C., Couteaudier M., Gouzil J., Richard L., Montange T., Betsem E., Rua R., Tobaly-Tapiero J., Lindemann D., Njouom R. (2018). Potent neutralizing antibodies in humans infected with zoonotic simian foamy viruses target conserved epitopes located in the dimorphic domain of the surface envelope protein. PLoS Pathog..

[73] Switzer W.M., Bhullar V., Shanmugam V., Cong M.E., Parekh B., Lerche N.W., Yee J.L., Ely J.J., Boneva R., Chapman L.E. (2004). Frequent simian foamy virus infection in persons occupationally exposed to nonhuman primates. J. Virol..

[74] Switzer W.M., Salemi M., Shanmugam V., Gao F., Cong M.E., Kuiken C., Bhullar V., Beer B.E., Vallet D., Gautier-Hion A. (2005). Ancient co-speciation of simian foamy viruses and primates. Nature.

[75] Falcone V., Schweizer M., Neumann-Haefelin D. (2003). Replication of primate foamy viruses in natural and experimental hosts. Curr. Top. Microbiol. Immunol..

[76] Rua R., Betsem E., Montange T., Buseyne F., Gessain A. (2014). In vivo cellular tropism of gorilla simian foamy virus in blood of infected humans. J. Virol..

[77] Weingartl H.M., Embury-Hyatt C., Nfon C., Leung A., Smith G., Kobinger G. (2012). Transmission of Ebola virus from pigs to non-human primates. Sci. Rep..

[78] Zeng X., Blancett C.D., Koistinen K.A., Schellhase C.W., Bearss J.J., Radoshitzky S.R., Honnold S.P., Chance T.B., Warren T.K., Froude J.W. (2017). Identification and pathological characterization of persistent asymptomatic Ebola virus infection in rhesus monkeys. Nat. Microbiol..

[79] Warren T.K., Kane C.D., Wells J., Stuthman K.S., Van Tongeren S.A., Garza N.L., Donnelly G., Steffens J., Gomba L., Weidner J.M. (2021). Remdesivir is efficacious in rhesus monkeys exposed to aerosolized Ebola virus. Sci. Rep..

[80] Brinton M.A., Di H., Vatter H.A. (2015). Simian hemorrhagic fever virus: Recent advances. Virus Res..

[81] Lapin B.A., Shevtsova Z.V. (2015). [To the 50th anniversary of the discovery of the simian hemorrhagic fever and SHF virus]. Vopr. Virusol..

[82] Vatter H.A., Donaldson E.F., Huynh J., Rawlings S., Manoharan M., Legasse A., Planer S., Dickerson M.F., Lewis A.D., Colgin L.M. (2015). A simian hemorrhagic fever virus isolate from persistently infected baboons efficiently induces hemorrhagic fever disease in Japanese macaques. Virology.

[83] Lauck M., Sibley S.D., Hyeroba D., Tumukunde A., Weny G., Chapman C.A., Ting N., Switzer W.M., Kuhn J.H., Friedrich T.C. (2013). Exceptional simian hemorrhagic fever virus diversity in a wild African primate community. J. Virol..

[84] Wahl-Jensen V., Johnson J.C., Lauck M., Weinfurter J.T., Moncla L.H., Weiler A.M., Charlier O., Rojas O., Byrum R., Ragland D.R. (2016). Divergent Simian Arteriviruses Cause Simian Hemorrhagic Fever of Differing Severities in Macaques. Mbio.

[85] Kovacs G.M., Harrach B., Zakhartchouk A.N., Davison A.J. (2005). Complete genome sequence of simian adenovirus 1: An Old World monkey adenovirus with two fiber genes. J. Gen. Virol..

[86] Kobler H., Regenfuss P. (1971). [A simple technique for orotracheal intubation in the rabbit]. Z. Gesamte Exp. Med. Einschl. Exp. Chir..

[87] Morgan T.J., Glowaski M.M. (2007). Teaching a new method of rabbit intubation. J. Am. Assoc. Lab. Anim. Sci..

[88] Han J.W., La T.M., Kim J.H., Choi I.S., Song C.S., Park S.Y., Lee J.B., Lee S.W. (2018). The possible origin of human adenovirus type 3: Evidence of natural genetic recombination between human and simian adenovirus. Infect. Genet. Evol..

[89] Wevers D., Metzger S., Babweteera F., Bieberbach M., Boesch C., Cameron K., Couacy-Hymann E., Cranfield M., Gray M., Harris L.A. (2011). Novel adenoviruses in wild primates: A high level of genetic diversity and evidence of zoonotic transmissions. J. Virol..

[90] Morris S.J., Sebastian S., Spencer A.J., Gilbert S.C. (2016). Simian adenoviruses as vaccine vectors. Future Virol..

[91] O’Sullivan M.G., Anderson D.C., Fikes J.D., Bain F.T., Carlson C.S., Green S.W., Young N.S., Brown K.E. (1994). Identification of a novel simian parvovirus in cynomolgus monkeys with severe anemia. A paradigm of human B19 parvovirus infection. J. Clin. Investig..

